# Supplementary material for: DNA twist at high alkali ion concentrations: evidence against C-form DNA in solution
Source: Nucleic Acids Res. 2026 Mar 12;54(5):gkag192. doi: 10.1093/nar/gkag192 (PMC12980068; doi:10.1093/nar/gkag192)
Supplement: gkag192_Supplemental_File [file gkag192_supplemental_file.pdf]

SUPPLEMENTARY INFORMATION FOR

DNA twist at high alkaline ion concentrations: Evidence against C-form DNA in solution

Koen R. Storm[1,2,†], Christian Wiebeler[2,†], Sergio Cruz-León[3], Caroline Körösy[1,2], Nadine Schwierz[2,*], and Jan Lipfert[1,2,*]

[1]Soft Condensed Matter and Biophysics, Department of Physics and Debye Institute for Nanomaterials Science, Utrecht University, Princetonplein 1, 3584 CC Utrecht, The Netherlands
[2]Institute for Physics, University of Augsburg, Universitätsstrasse 1, 86159 Augsburg, Germany
[3]Department of Theoretical Biophysics, Max Planck Institute of Biophysics, Max-von-Laue-Straße 3, 60438 Frankfurt am Main, Germany.

[†]Equal contribution
*Correspondence: Nadine.Schwierz@uni-a.de; Jan.Lipfert@uni-a.de

<u>**Content**</u>

Supplementary Methods

Supplementary Tables S1-S5

Supplementary Figures S1-S26

Supplementary References

Supplementary Methods

Determination of Activity Derivative from Simulations and Experiments

The activity coefficient a_c is a factor to take the experimentally measured deviation of a mixture from ideal behavior into account. Similarly, the activity derivative a_{cc} provides information on the balance between ion-ion and ion-water interactions (1,2). Kirkwood-Buff (KB) theory (3) was employed to compute the a_{cc} of LiCl at various concentrations. Based on radial distribution functions between two species i and j, the KB integrals G_{ij} allow to determine a variety of thermodynamic properties (4):

$$G_{ij} = 4\pi \int_0^\infty \left[g_{ij}^{\mu VT}(r_{ij}) - 1\right] r_{ij}^2 \, dr_{ij}, \tag{1}$$

with $g_{ij}^{\mu VT}(r_{ij})$ being the radial distribution function of the two species i and j in the grand canonical ensemble and r_{ij} being the center of mass distance between the two. Formally, the KB integrals are defined for infinite space and open systems (µVT), but MD simulations are typically realized for closed systems (NVT, NpT). Furthermore, due to the finite size of simulation boxes, the integration cannot be carried out over all space. Therefore, the KB integrals are truncated and rescaled resulting in:

$$G_{ij} \approx 4\pi \int_0^R \left[g_{ij}^{NpT}(r'_{ij}\rho) - 1\right] r_{ij}^2 \, dr_{ij}, \tag{2}$$

in which the radial distribution function is rescaled via $g_{ij}^{NpT}(r'_{ij}\rho) = f(\rho) g_{ij}^{sim}(r_{ij})$ with a prefactor $f(\rho)$ that is adjusted to ensure the correct asymptotic behavior at large distances (5).

The activity derivative a_{cc} is defined as:

$$a_{cc} = \left(\frac{\partial \ln a_c}{\partial \ln \rho_c}\right)_{p,T} = 1 + \left(\frac{\partial \ln \gamma_c}{\partial \ln \rho_c}\right)_{p,T} = \frac{1}{1 + \rho_c(G_{cc} - G_{co})}, \tag{3}$$

with the activity $a_c = \rho_c \gamma_c$, the cosolvent molar activity coefficient γ_c, and number density ρ_c.

For monovalent cations and anions, the required expressions are (2):

$$G_{cc} = \frac{1}{4}[G_{++} + G_{--} + 2G_{+-}] \text{ and } G_{co} = G_{oc} = \frac{1}{2}(G_{+o} + G_{-o}),$$

where +, -, and o denote cation, anion, and water oxygen, respectively.

For the simulations of LiCl with Mamatkulov-Schwierz parameters (2) in water at various concentrations, we employed cubic boxes with TIP3P water molecules (6) and inserted the corresponding numbers of cations and anions by replacing water molecules (**Supplementary Table S4**). The number of ions required for a given concentration was determined based on the number of water molecules in the simulation box. The equilibration protocol consisted of energy minimization followed by NVT and NpT equilibration. Energy minimization with the steepest descent algorithm used a maximum of 50000 steps. Subsequently, we used 1 ns of NVT and 1 ns of NpT simulations for further equilibration. In these simulations, the temperature of 300 K was maintained using the Berendsen thermostat with a coupling constant of 0.1 ps. In the NpT simulations, the isotropic Berendsen barostat with a coupling constant of 1 ps was employed to ensure a pressure of 1 atm.

For production runs, we used again NpT simulations at 300 K and 1 atm, but now by employing the velocity rescaling thermostat with a coupling constant of 0.1 ps and the isotropic Parrinello-Rahman barostat with a coupling constant of 5 ps. Three independent production simulations were carried out for 150 ns each. For determining the radial distribution functions, the first 5 ns of each production was discarded for equilibration. Furthermore, the radial distribution functions between ions were normalized in such a way that their average at large distances is 1. The KB integrals were evaluated numerically by employing the trapezoidal rule and with these integrals the activity derivative was determined via the final expression in Eq. (3). The three values for a_{cc} per concentration were then used to evaluate the average and the standard error of the mean.

For obtaining experimental values of a_{cc} for LiCl, we started with a selection of experimental activity coefficients taken from Hamer and Wu (7). First, the concentrations reported in molality were converted into molarity by employing a parabolic fit for molarity as a function of molality based on reference values (8) (**Supplementary Figure S24A**). To better approximate the required derivative, we performed a cubic fit of the activity coefficients as function of molarity in the concentration range from 0.09 to 8.40 M (**Supplementary Figure S24B**). We employed the equation of the cubic fit to generate closely spaced data points for $\ln \rho_c$ and $\ln a_c$ in the concentration range from 0.09 to 8.40 M (i.e. up to a molality of around 10 mol/kg) and used the central difference scheme for obtaining a_{cc}. These values for the concentrations of interest are shown in **Supplementary Figure S24C** and **Figure 5A**. The same approach was also employed to obtain a_{cc} for NaCl in the concentration range from 0.09 to 5.50 M. The corresponding values for a_{cc} as a function of concentration are shown in **Supplementary Figure S25C**.

Results from Simulations with Different Water Models

In addition to the assessment of DNA force fields in the manuscript and **Supplementary Figure S16**, we also tested the influence of the water model. For this purpose, we performed simulations with the OPC water model, but with otherwise identical force fields for DNA and ions as used in the parmbsc1 simulations of the manuscript, i.e. parmbsc1/Mamatkulov-Schwierz (**Supplementary Figure S18**). The 4-site OPC model is one of the more recent promising water models, as it reproduces liquid-water electrostatics and further physical properties of water better than TIP3P (9,10). In addition, we also include the results from the solvent parameterization of Strelnikov et al. in this discussion, as it employs the 4-site TIP4P-Ew water model (11) with the Joung-Cheatham ion parameters (12) combined with the same parmbsc1 force field for DNA.

Similar to all other combinations of force fields, we observe a quick drop in helical twist for simulations that started in C-DNA conformation for LiCl concentrations of 1.0, 4.4, and 7.8 M for the OPC water simulations (**Supplementary Figure S21**). After the initial equilibration, the twist from simulations with both starting conformations fluctuates around similar values. The averaged values for ΔTw at 1.0 M LiCl found with the OPC water model are similar to the values obtained with TIP3P and to the experimental data (**Supplementary Figure S18A**). However, the change in twist at higher concentrations is underestimated in the simulations using the OPC water model. For 4.4 M concentration, ΔTw from the parameterization by

Strelnikov et al. and with the OPC water model are similar and underestimate the results from experiments and simulations with TIP3P. Similarly, the OPC simulations at 7.8 M concentration underestimate the increase in twist relative to the simulations using TIP3P water and compared to the experiments. The absolute twist values from the solvent parameterization of Strelnikov et al. are higher than the ones from the other two force fields (**Supplementary Figure S18B**), but this effect cancels for the change in twist, as with this parameterization the 0.1 M KCl reference simulations also exhibit the largest twist.

Overall, the combination of parmbsc1 with the more recent water model OPC appears to be a compromise, as the water properties are better reproduced, but DNA twist is less well described. For this study, we focused on DNA properties at high salt concentration and for that purpose parmbsc1 with TIP3P yields the best agreement with experiments.

Assessment of Longer Simulation Times

In the simulations reported in the manuscript, we employed -after a sufficiently long equilibration- three independent 300 ns production simulations for each concentration and DNA conformation. The change in twist (ΔTw) obtained with this approach at 1 M concentration is in good agreement with our previous study, where a single trajectory was propagated for 3 μs simulation time (**Figure 3B**). To test convergence, we extended our parmbsc1 simulations from the highest concentration of 7.8 M. For this purpose, we continued each of the six productions to 1.2 μs and discarded the first 100 ns of these simulations from analysis. In all cases, ΔTw is virtually the same independent of whether the simulation started in B- or C-Form (**Supplementary Figure S19**). Importantly, we do not see significant differences in the simulated ΔTw upon extension of our simulations up to 1.2 μs, as assessed by two-sided Welch's t-tests for the different time intervals (**Supplementary Figure S19**). We conclude that 3 x 300 ns production run after sufficient equilibration (energy minimization + 1 ns NVT + 301 ns NPT) is sufficient to yield converged results.

Influence of DNA Duplex Length on Change in Twist

To test how the sequence and length of the simulated dsDNA affects the observed ΔTw, we carried out control calculations based on our simulations of the 33 bp DNA duplex and determined ΔTw for different duplex lengths by removing more and more bps at each end of the simulated helix (**Supplementary Figure S20**). We find that similar changes in DNA twist are obtained when different length DNA segments are analyzed. Our control calculations suggest that discarding the first and last three bps (as we report in the main text of the paper) is sufficient to obtain stable results and also indicates that our findings are insensitive to changes in DNA length and sequence, consistent with previous findings (13).

Results from Simulations with NaCl

We deliberately chose Li^+ as cation, as it causes the largest increase in twist in experiments. We also performed additional simulations with NaCl at 1.0 and 4.4 M concentrations with parmbsc1/Mamatkulov-Schwierz parameters and TIP3P water model. Furthermore, we included data from our previous publication (13), where the same parameterization was employed.

Similar to the LiCl simulations at high salt concentration (**Figure 4A**), the absolute twist of the structure that started in C-form quickly decays to a value typical for the B-form and after that, the twists from simulations of both forms fluctuate around similar values (**Supplementary Figure 22**). Furthermore, the change in twist as a function of concentration is in good agreement with experiments up to 1 M (**Supplementary Figure 23A**). However, at 4.4 M the results clearly deviate from the experiments. As for LiCl, the deviation results from limitations in the activity derivative at high concentration. Specifically, it is not possible to increase the activity derivative in simulations beyond 1.0 and reach the experimentally relevant regime where saturation sets in (**Supplementary Figure 23B,C**). Moreover, there is not a monotonous increase of the activity derivative with increasing concentration in contrast to the experiments. This highlights the importance of accurate ion-ion interactions and the necessity to improve the NaCl force fields to correctly describe the DNA conformation at high salt concentrations.

Supplementary Tables

Supplementary Table S1. Fitting parameters for second-degree polynomial fits to the experimental index of refraction n vs. salt concentration x (in M) data (Supplementary Figure S2B).

Model	Salt	Parameter	Value
$n=ax^2+bx+c$	LiCl	a	-0.0000922
		b	0.00866
		c	1.33296
	NaCl	a	-0.0002123
		b	0.00977
		c	1.33308
	KCl	a	-0.0002090
		b	0.00965
		c	1.33306
	CsCl	a	-0.0001123
		b	0.01247
		c	1.33318

Supplementary Table S2. Difference in the molar absorption at 280 nm as a function of LiCl concentrations. Data are from the CD spectra in **Figure 2**.

[LiCl] (M)	$\Delta\varepsilon_{280nm}$ (M^{-1} cm^{-1})
0.1	2.42
1	1.61
2	0.97
4	0.16
6	-0.41
8	-0.89

Supplementary Table S3. Number of water molecules, cations and anions for the simulations of double-stranded DNA starting in B- or C-form, respectively, for the simulations with the parmbsc1 force field. Furthermore, the average volume from the production simulations and the resulting molarity are listed.

B-Form					
Molality (m)	# H_2O	# Li^+	# Cl^-	Volume (nm^3)	Molarity (M)
1.00	66941	1271	1207	2089.5	0.96
2.00	64692	2396	2332	2061.0	1.88
3.00	62588	3448	3384	2037.6	2.76
4.00	60618	4433	4369	2018.3	3.59
5.01	58766	5359	5295	2002.3	4.39
6.01	57026	6229	6165	1988.9	5.15
7.01	55384	7050	6986	1977.5	5.87
8.01	53834	7825	7761	1967.6	6.55
9.01	52370	8557	8493	1958.8	7.20
10.01	50982	9251	9187	1950.8	7.82
**C-Form**					
Molality (m)	# H_2O	# Li^+	# Cl^-	Volume (nm^3)	Molarity (M)
1.00	64997	1235	1171	2029.3	0.96
2.00	62811	2328	2264	2001.6	1.88
3.00	60769	3349	3285	1978.9	2.76
4.00	58855	4306	4242	1960.1	3.59
5.01	57057	5205	5141	1944.6	4.39
6.01	55367	6050	5986	1931.6	5.15
7.01	53773	6847	6783	1920.5	5.86
8.01	52269	7599	7535	1910.9	6.55
9.01	50847	8310	8246	1902.3	7.20
10.01	49501	8983	8919	1894.5	7.82

Supplementary Table S4. Number of water molecules, cations and anions for the simulations of ions in water (without DNA) to determine the activity derivatives. Furthermore, the average volume from the production simulations and the resulting molarity are listed.

**LiCl in Water**					
Molality (m)	# H_2O	# Li^+	# Cl^-	Volume (nm^3)	Molarity (M)
0.10	2157	4	4	65.66	0.10
0.26	2145	10	10	65.47	0.25
0.52	2125	20	20	65.17	0.51
1.04	2087	39	39	64.64	1.00
2.01	2019	73	73	63.77	1.90
3.02	1953	106	106	63.03	2.79
4.02	1891	137	137	62.42	3.64
5.00	1835	165	165	61.94	4.42
6.03	1779	193	193	61.50	5.21
7.00	1729	218	218	61.15	5.92
8.00	1681	242	242	60.84	6.60
9.00	1635	265	265	60.56	7.27
10.02	1591	287	287	60.30	7.90

Supplementary Table S5. Correlations of helical properties with helical twist, activity derivative (a_{cc}), and number of adsorbed ions (# Ions). The helical properties consisted of the six base-pair step parameters with respect to a local helical frame (x-displacement, y-displacement, helical rise, inclination, tip, helical twist), major and minor groove widths and radius as defined in (14) as well as the average pucker and number of adsorbed ions (# Ions). Correlations are sorted in decreasing order of the absolute value of the correlation coefficient.

**Helical Twist ΔTw**		**a_{cc}**		**# Ions**	
Property	R value	Property	R value	Property	R value
# Ions	0.960	Helical Rise	-0.976	Helical Twist	0.960
Major Groove	-0.958	Helical Twist	0.924	Major Groove	-0.937
Radius	-0.951	Minor Groove	-0.870	Helical Rise	-0.901
Helical Rise	-0.947	Radius	-0.864	Radius	-0.877
x-Displacement	0.934	# Ions	0.859	x-Displacement	0.867
Pucker	0.847	Major Groove	-0.858	Pucker	0.821
Minor Groove	-0.777	x-Displacement	0.850	Minor Groove	-0.697
y-Displacement	0.359	Pucker	0.779	Inclination	0.430
Inclination	0.353	y-Displacement	0.298	y-Displacement	0.406
Tip	0.146	Inclination	0.252	Tip	0.159
		Tip	0.089		

Supplementary Figures

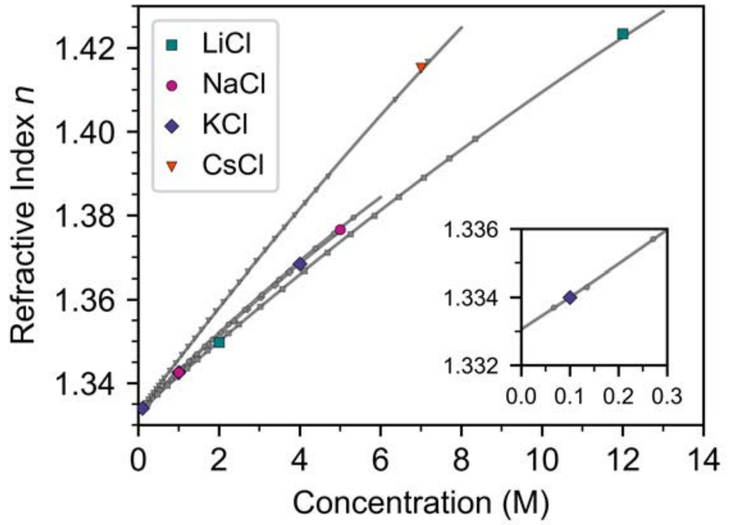

Supplementary Figure S1. Concentration determination of salt stocks by index of refraction measurements. We determined the index of refractions of several of our salt solutions using and Abbe refractometer (λ=589.3 nm, 21 ºC; Abbe Refractometer 3T, Atago). Measured data from this work are shown as colored symbols at the nominal concentration the stocks were prepared at by weighing the salts. Our measurements are in excellent agreement with the expected indices of refraction shown as grey symbols taken from (8). The solid lines are second-degree polynomial fit to the literature data.

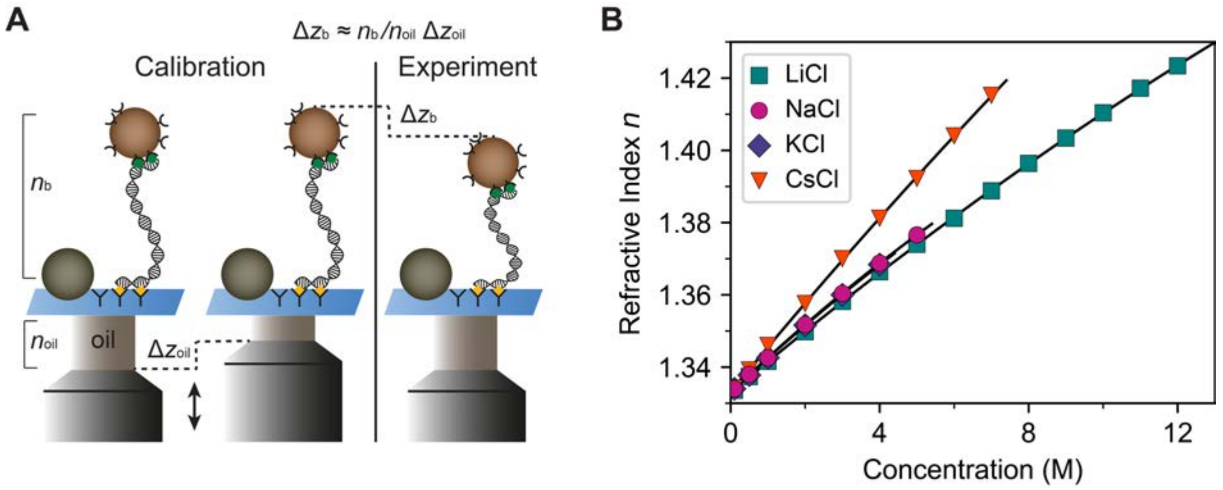

Supplementary Figure S2. Effect of the index of refraction on MT measurements. A) Schematic of the vertical displacement determination technique of magnetic tweezers. Prior to the experiment, calibration is performed where diffraction patterns are collected while vertically displacing the oil objective, i.e., a displacement of Δz_{oil}. During measurements, the vertical position of the bead is correlated to the diffraction pattern. The absolute change in the vertical position depends on the ratio of the refractive index of the oil n_{oil} and of the buffer n_{b}. We determine the absolute Δz_{b} according to $\Delta z_{\mathrm{b}} = n_{\mathrm{oil}} / n_{\mathrm{b}} \cdot \Delta z_{\mathrm{oil}}$. B) Refractive index of salt solutions in 10 mM Tris. The solid lines are second-degree polynomial fits to the data with coefficients shown in **Supplementary Table S1**.

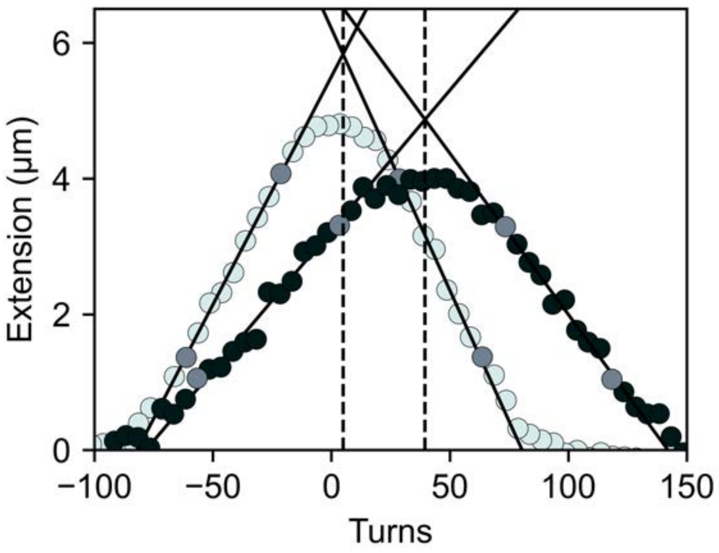

Supplementary Figure S3. Determination of change in twist from MT rotation curves. Extension vs. applied turns measurements for our 20.6 kbp DNA construct at an applied force of 0.25 pN for the 0.1 (light data points) and 7 M (black data points) LiCl conditions. In the plectonemic regime past the buckling points, the DNA tether shows a linear decrease in extension vs. turns upon addition of additional of positive or negative supercoils, respectively. The slopes in the buckling regimes at positive and negative turns are determined by fitting a linear relationship to the data in this range (grey circles indicate the beginning end of the fitting range), as described in Ref. (15). The centers of the rotation curves are determined as the intersection of the extrapolated fitted lines.

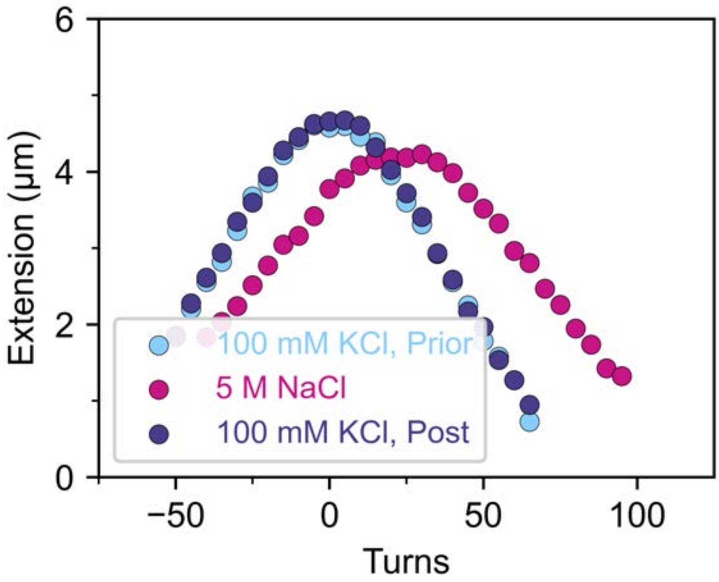

Supplementary Figure S4. Reversibility of twist increase. Rotation-extension curves collected in the 100 mM KCl reference condition and in 5 M NaCl. Upon return to the reference condition by exchanging 5 M NaCl for 100 mM KCl, the initial rotation curve is recovered within experimental error.

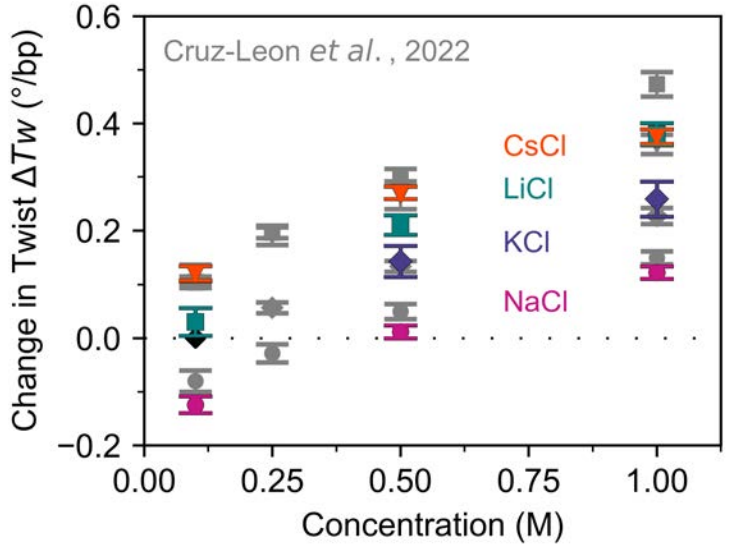

Supplementary Figure S5. Comparison of MT twist measurements with Cruz-Leon *et al.* for different monovalent ions. Changes in DNA twist determined from MT measurements as described in Materials and Methods and Figure 1. Grey data are from Cruz-Leon *et al.* (13) using a 7.9 kbp DNA construct with a GC content of 56%. Colored data points are from this work (same data as in Figure 1) using a 20.6 kbp DNA construct with a GC content of 46%; data points indicate the mean ± SD from at least 4 molecules. 100 mM KCl was used as a reference condition throughout. Overall, the data show good reproducibility. The data from this work and Cruz-Leon *et al.* for both CsCl (triangles) and KCl (diamonds) are within experimental error. For LiCl (squares) and NaCl (circles), we find a slightly, but systematically lower twist relative to 100 mM KCl in this work compared to the data of Cruz-Leon *et al.,* which could be due differences in the effects of salts on DNA twist for constructs with different GC content (**see Supplementary Figure S6**).

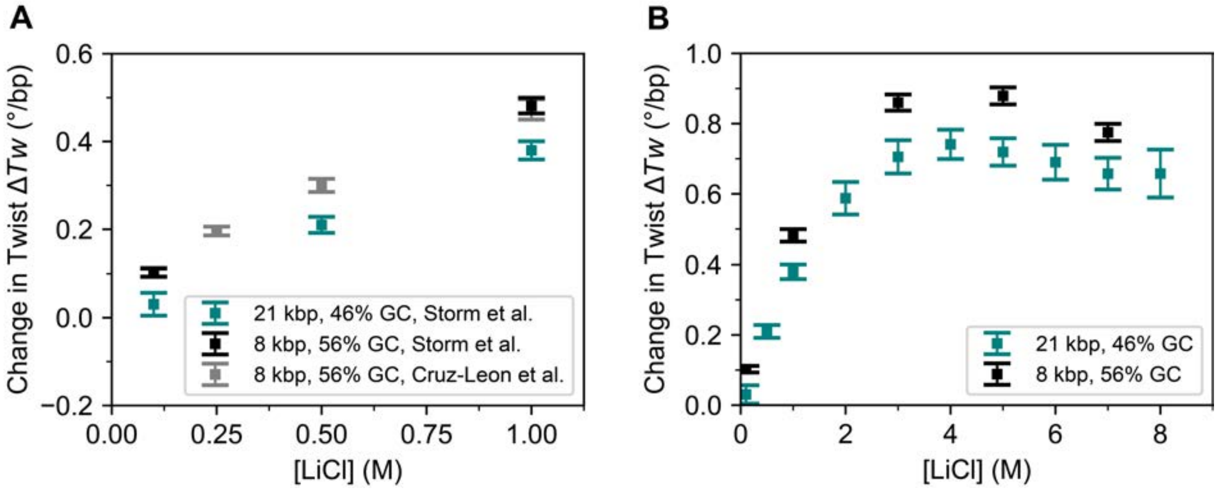

Supplementary Figure S6. Comparison of MT twist measurements for different DNA constructs as a function of LiCl concentration. A) Changes in DNA twist determined from MT measurements as described in Materials and Methods and Figure 1. Dark green data points are from this work (same data as in Figure 1) using a 20.6 kbp DNA construct with a GC content of 46%; data points indicate the mean ± SD from at least 18 molecules. Black data points are from this work using a 7.9 kbp DNA construct with a GC content of 56%; data points indicate the mean ± SD from at least 12 molecules. Grey data are from Cruz-Leon et al. (11) using the same 7.9 kbp DNA construct with a GC content of 56%. 100 mM KCl was used as a reference condition throughout. B) Changes in DNA twist determined from MT measurements. Same color scheme as panel A. Data points indicate the mean ± SD from at least 11 molecules.

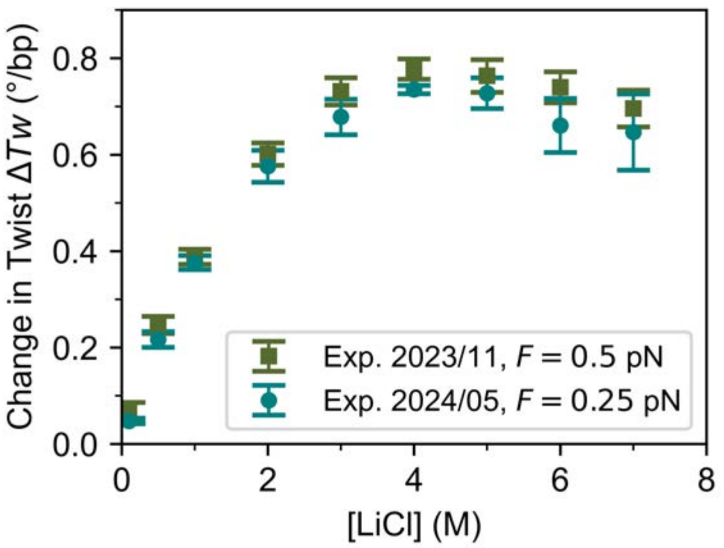

Supplementary Figure S7. Reproducibility of MT measurements of DNA twist. Changes in DNA twist determined from MT measurements as described in Materials and Methods and Figure 1. Comparison of a first LiCl data set, from a 10 M LiCl stock (≥ 99%, Sigma-Aldrich), recorded in 10 mM Tris pH 7.6 and using rotation curves at $F = 0.5$ pN (2023/11), and a second data set, prepared as described in Materials and Methods, using rotation curves at $F = 0.25$ pN (2024/05). The two data sets recorded for this work are in excellent agreement, within experimental error. Symbols are the mean ± SD of at least 2 molecules.

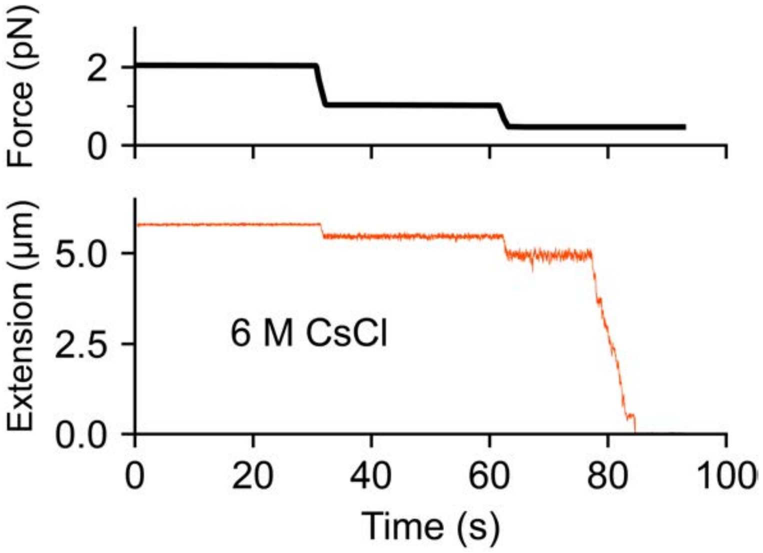

Supplementary Figure S8. Collapse of DNA in the presence of 6 M CsCl. Extension time trace of a 20.6 kbp DNA tether in 6 M CsCl solution in 10 mM Tris HCl pH 7. The applied force is indicated in the top panel. A collapse of the DNA tether is observed in the 6 M CsCl solution as the stretching force is lowered from 2 pN to 0.5 pN.

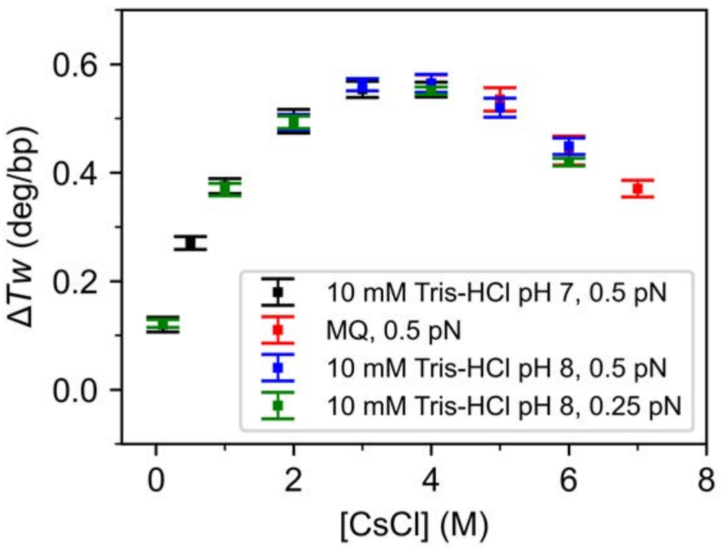

Supplementary Figure S9. Effect of buffer condition and force on MT twist measurements for CsCl solutions. Change in DNA twist as a function of CsCl concentration relative to 100 mM KCl with 10 mM Tris-HCl pH 7 for different buffers, obtained from rotation curves (Figure 1) recorded at forces of either 0.25 or 0.5 pN. MT measurements revealed occasional collapse of DNA molecules in 10 mM Tris HCl pH 7 at both 0.25 pN and 0.5 pN, which restricted the concentration range probed to 4 M. We therefore performed MT measurement in the absence of buffer (in milliQ water, MQ) to exclude the role of additional ions. In the absence of buffer, we were able to expanded the concentration range to the solubility limit of 7 M CsCl. However, repeat measurements still resulted in DNA collapse. Therefore, to increase the buffering capacity and to investigate the role of controlled pH, we probed CsCl concentrations in 10 mM Tris HCl pH 8 which enabled consistent repeat measurements at both 0.25 pN and 0.5 pN without the observation of DNA collapse. Overall, regardless of buffering condition, the change in DNA twist observed as a function of CsCl concentration is consistent within error.

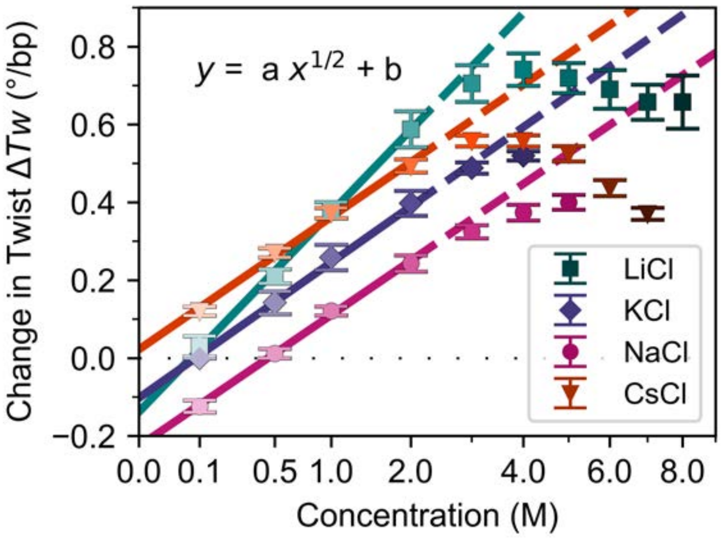

Supplementary Figure S10. Changes in DNA twist with salt concentration as determined from MT measurements shown on a linear-logarithmic scale. The symbols show the change in DNA twist determined experimentally (same data as Figure 1G). The solid lines indicate square-root dependencies fitted up to 2 M. Dashed lines are the extrapolation of the fits beyond a concentration of 2 M. The data are well described by the square root dependencies up to 2 M. At higher salt concentrations deviations from the square-root scaling are apparent and the experimental data fall below the square root trend lines.

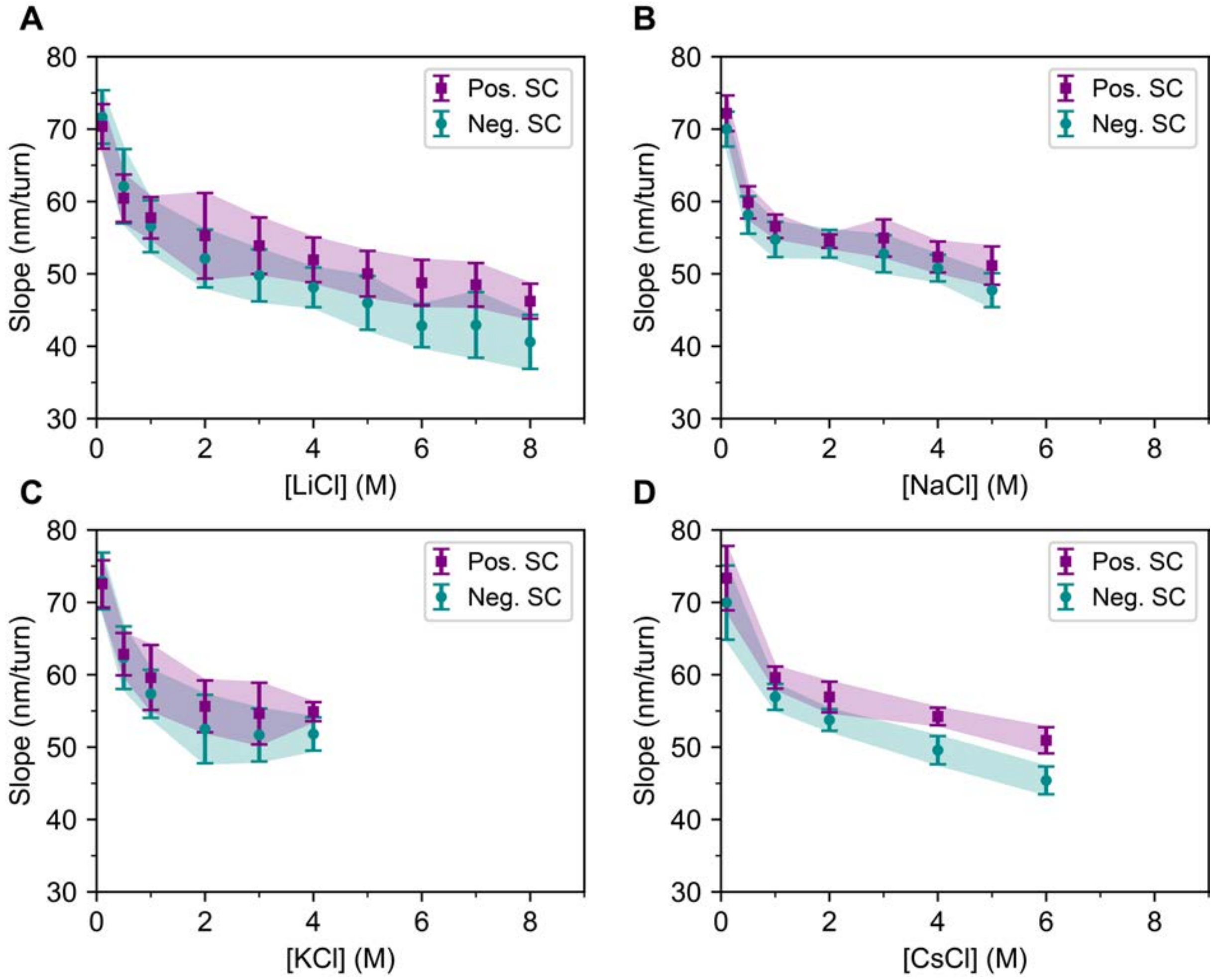

Supplementary Figure S11. Fitted slopes of DNA extension vs. applied rotation in the presence of LiCl, NaCl, KCl or CsCl. Extension vs. turns slopes in the plectonemic regime from fits to the rotation curve data for different concentrations of A) LiCl (same data as Figure 1B), B) NaCl, C) KCl, or D) CsCl recorded at a force $F = 0.25$ pN.

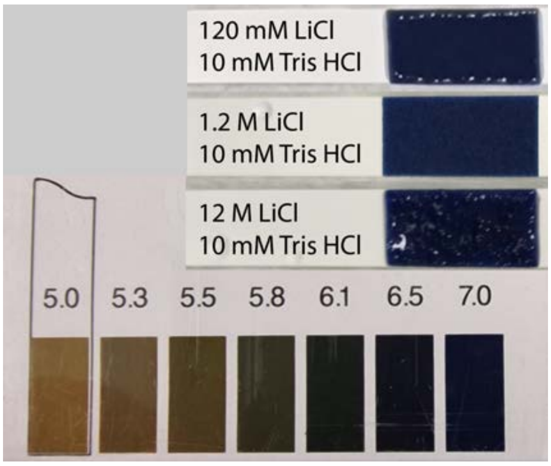

Supplementary Figure S12. Measurements of solution pH at different LiCl concentrations. Measurements of pH for a broad range of LiCl concentrations using pH indicator paper (Merck, Germany). Aqueous LiCl has been shown to remain close to neutrality but suggested to cause slight acidity at extreme conditions (16). The pH indicator strips show that solutions up to 12 M do not present noticeable acidity under our conditions.

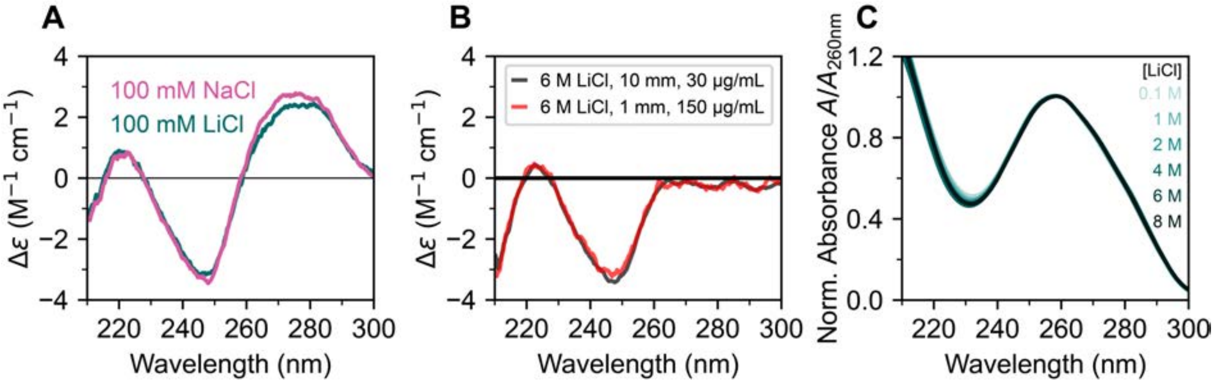

Supplementary Figure S13. Circular Dichroism of DNA in varying salt concentrations. A) CD spectra of λ-DNA in 100 mM NaCl and 100 mM LiCl. Slight ion specificity is observed as the magnitude of the band at 280 nm appears larger for NaCl than for LiCl. B) CD spectra of λ-DNA in 6 M LiCl solution for DNA concentrations of 30 µg/mL and 150 µg/mL (measured using 10 mm or 1 mm pathlength cuvettes, respectively, to achieve similar levels of signal-to-noise and to keep the absorption in a reasonable range). The resulting spectra are essentially within error, suggesting that intermolecular interactions do not significantly influence the CD spectra under our conditions. C) Normalized absorbance of λ-DNA for LiCl concentrations of 0.1-8 M. The spectra are essentially superimposable, indicating again the absence of formation of large DNA aggregates.

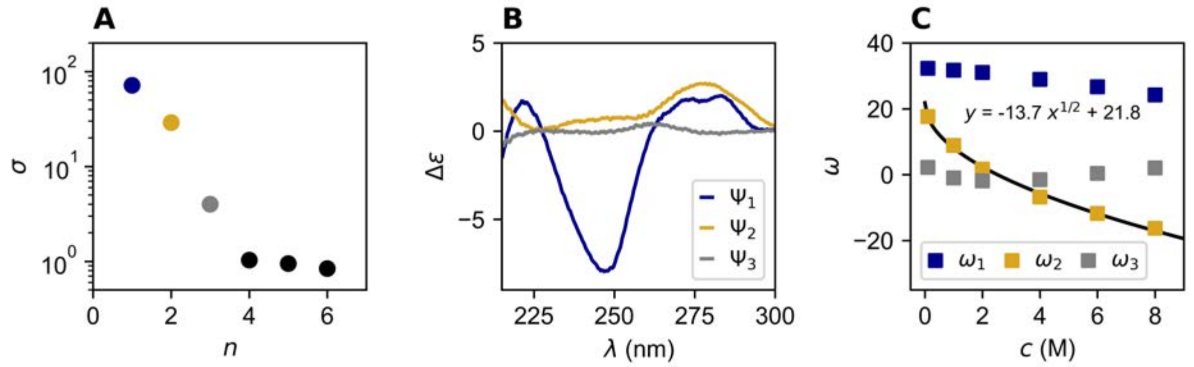

Supplementary Figure S14. Singular value decomposition of CD spectra. We applied analysis by singular value decomposition (SVD) to the CD spectra of λ-DNA (Figure 2) in varying concentrations of LiCl. SVD was implemented by representing the i-th observed CD spectrum $\{\overrightarrow{\Phi_i} | 1 \leq i \leq n\}$ of the DNA in salt condition i as a vertical vector of dimension m measured for the wavelengths channels $\{\lambda_j | 1 \leq j \leq m\}$. The matrix M comprises all spectral vectors and is a m×n rectangular matrix where m > n.

The SVD transformation (17,18) decomposes the matrix of the CD spectra M as follows:

$$\mathrm{M} = \mathrm{U}DV^T = \mathrm{U}(\sigma_1 \overrightarrow{v_1} \quad \sigma_2 \overrightarrow{v_2} \ \ldots \ \sigma_r \overrightarrow{v_r}) = \mathrm{U}(\overrightarrow{\omega_1} \quad \overrightarrow{\omega_2} \ \ldots \ \overrightarrow{\omega_r}).$$

The matrix U consists of the basis function vectors Ψ as columns. The basis functions are not CD spectra themselves, but the spectra can be represented in the basis of the (orthogonal) Ψ_i. D is a diagonal n×n matrix that has the singular values σ_i on the diagonal. The product $DV^T = \vec{\omega}_i$ gives the (LiCl-dependent) weights of the basis functions Ψ_i when constructing the spectra from the basis of the Ψ_i.

A) Singular values σ obtained from the singular value decomposition of the six experimentally determined CD spectra as a function of LiCl concentration. Singular values σ indicate the relative importance of the basis functions for representing the data. While the first two components clearly have large singular values, the last three have small singular values and likely correspond to noise. The third singular value is somewhat intermediate. B) Basis functions Ψ for the three components with the highest σ values. The first two components clearly contain signal while the third component is somewhat marginal. C) Coefficients corresponding to the basis functions ω. The solid line is a fit of a square root dependance to the ω_2 values.

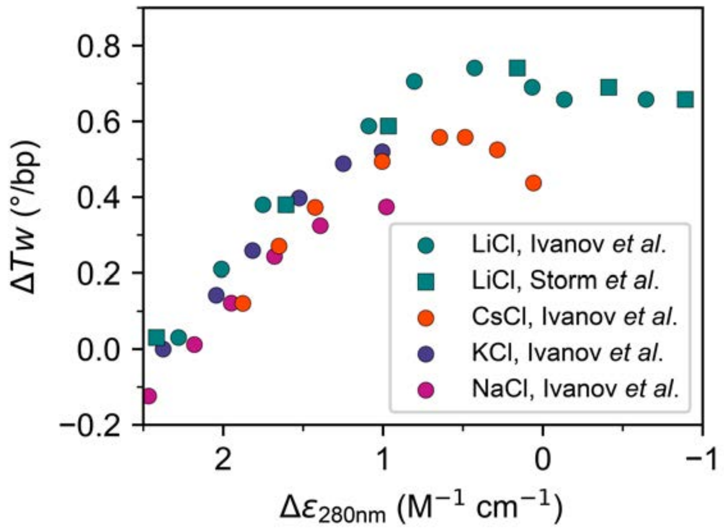

Supplementary Figure S15. Changes in DNA twist vs. CD values at 280 nm for different monovalent salts. The values for twist relative to the 100 mM KCl reference conditions were taken from the magnetic tweezers data obtained in this work (Table 1 and Figure 1G). Data for $\Delta\varepsilon_{280nm}$ were taken either from this work (green squares) or from the Ref. (19) (circles).

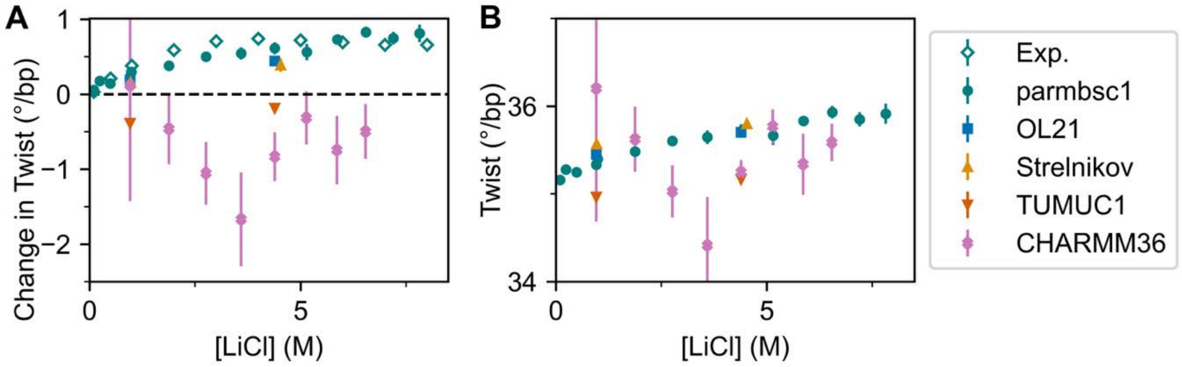

Supplementary Figure S16. DNA twist from MD simulations for different force fields as a function of LiCl concentration. A) Change in twist as a function of LiCl concentration relative to 0.10 M KCl from experiments and MD simulations with different force fields. A subset of the data are shown in Figure 3. The negative changes in twist from the simulations with TUMUC1 are caused by a too strong affinity of Li^{+} towards the oxygen or nitrogen atoms of the nucleobases (**Supplementary Figures S15A and S18B**). The CHARMM36 simulations show very large scatter, due to the unzipping of the DNA ends (**Supplementary Figure S15B**). B) Absolute values of the DNA twist as a function of LiCl concentration. Data correspond to the mean ± SEM from six independent simulations.

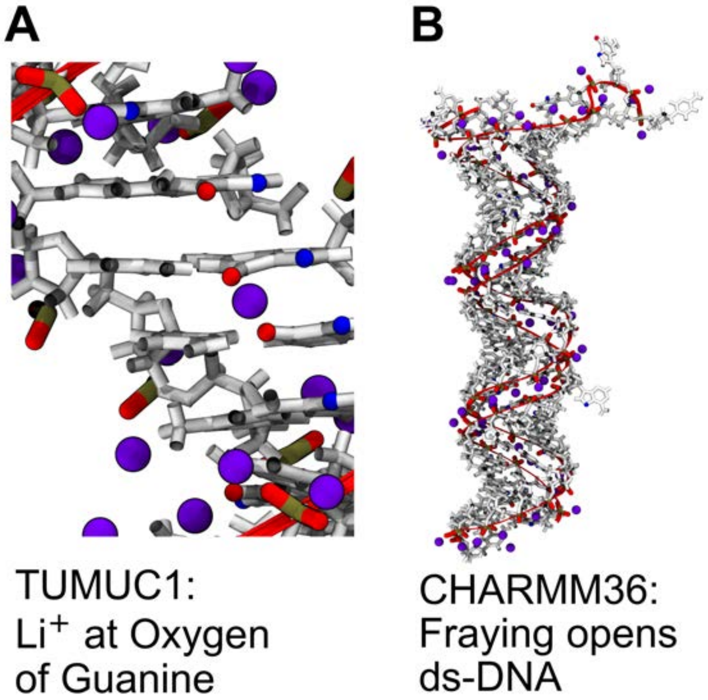

Supplementary Figure S17. Simulation snapshots illustrating typical structures from simulations with the TUMUC1 and CHARMM36 force fields. A) In case of TUMUC1, more Li^+ (purple) are adsorbed than with parmbsc1 (**Supplementary Figure S18)**. The strong adsorption involves the oxygen atoms of guanine, where the ions bridge stacked nucleobases. B) During the simulations with CHARMM36, the DNA ends unzip, resulting in unreliable values for the helical parameters. This opening is shown at the upper part of the depicted DNA structure, where one strand of the DNA backbone (red) is not paired with the other one.

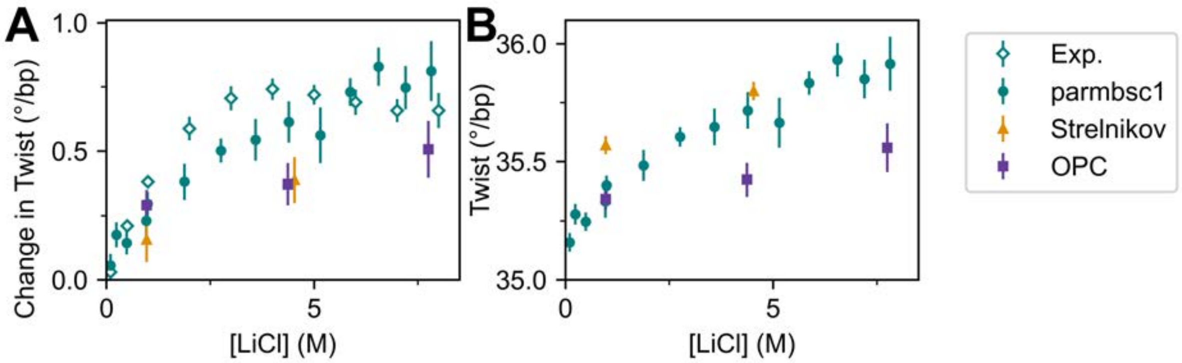

Supplementary Figure S18. DNA twist from MD simulations for different water models as a function of LiCl concentration. A) Change in twist as a function of LiCl concentration relative to 0.10 M KCl from experiments (open diamonds) and MD simulations for three force fields: parmbsc1/Mamatkulov-Schwierz with TIP3P water model (dark green diamonds), parmbsc1/Joung-Cheatham with TIP4P-Ew (orange triangles), and parmbsc1/Mamatkulov-Schwierz with OPC (purple squares). B) Absolute values of the DNA twist as a function of LiCl concentration for the same force fields. Data correspond to the mean ± SEM from six independent simulations.

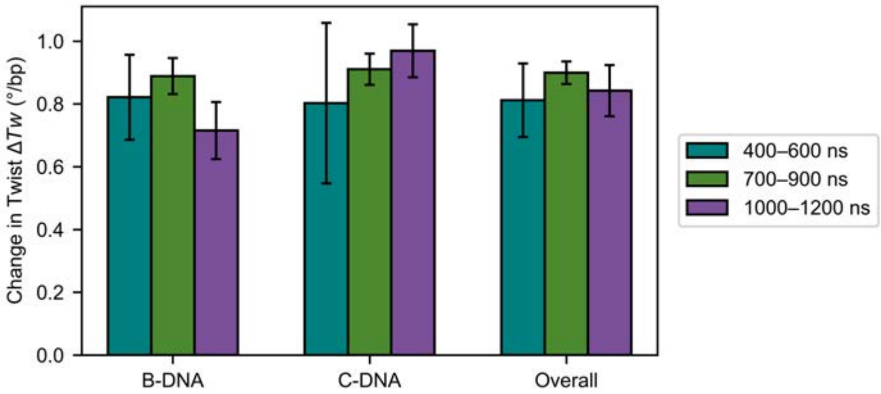

Supplementary Figure S19. DNA twist remains stable in µs-long MD simulations. The change in DNA twist (ΔTw) (with respect to the 100 mM KCl reference condition) from long, 1.2 µs total, control simulations in 7.8 M LiCl was averaged over the time windows indicated in the legend for trajectories either starting in B- or C-form or for both forms. The simulations were performed with the parmbsc1 force field for DNA, Mamatkulov-Schwierz ion parameters and TIP3P water model. Data correspond to mean ± SEM from three independent production runs for B- and C-DNA and from 6 in case of the last category. Comparing the results of the 1.2 µs simulations to the original 300 ns long runs, twist values are all within error and we observed no significant differences (all p-values are ≥ 0.45 as computed obtained for two-sided Welch's t-tests with unequal variances).

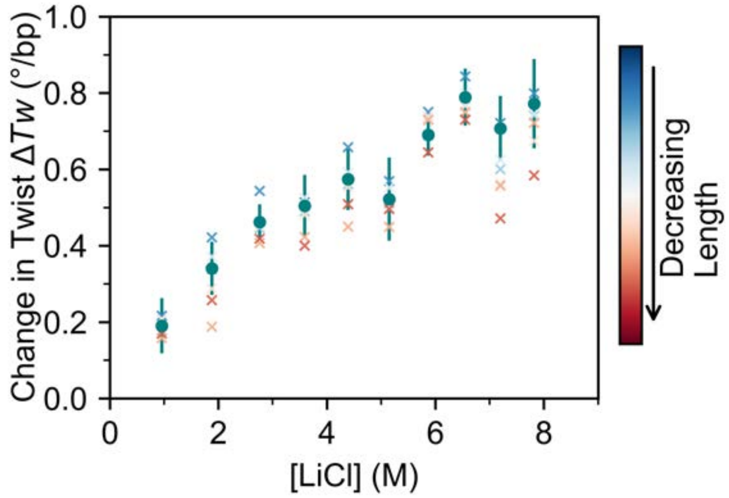

Supplementary Figure S20. Dependence of the change in DNA twist on duplex length. In addition to the values of ΔTw for the central 27 base pairs (bp) shown in the manuscript, we determined further values by leaving out more and more base pairs at both ends going down to 15 bps. Data points for 27 bps are shown as filled circles with mean ± SEM from six independent simulation runs. For the other six lengths, we only show the mean values as crosses omitting their uncertainties for clarity.

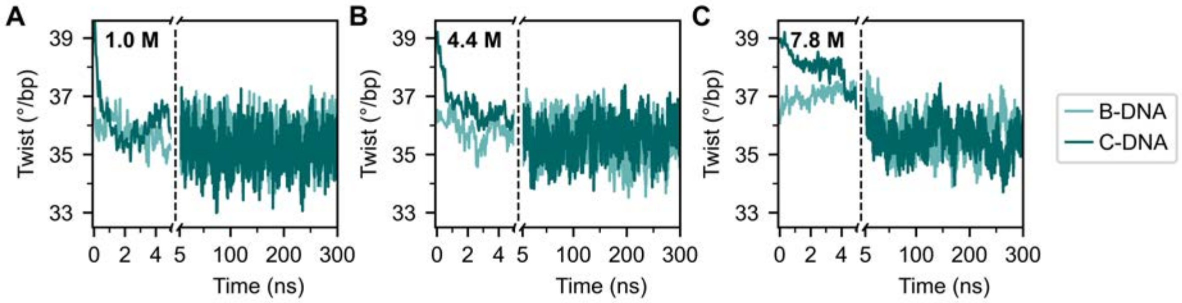

Supplementary Figure S21. C-DNA is unstable in three LiCl concentrations and converges to B-DNA in MD simulations with OPC water. Time series of twist at A) 1.0, B) 4.4 M, and C) 7.8 M LiCl initiated from an ideal B- and C-DNA structure. The simulations were performed with parmbsc1/Mamatkulov-Schwierz force fields and the OPC water model.

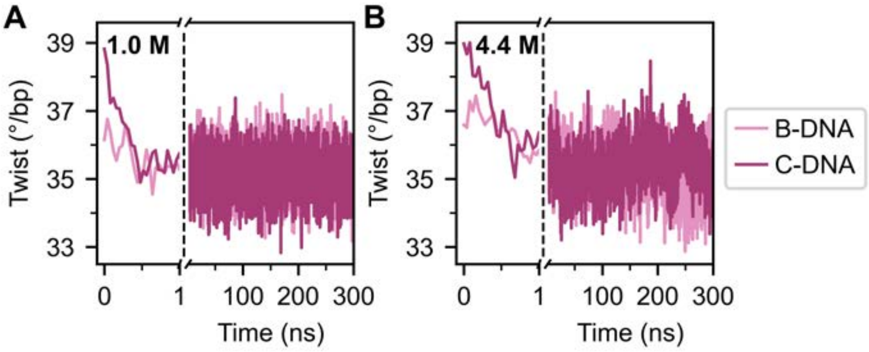

Supplementary Figure S22. C-DNA is unstable and converges to B-DNA in MD simulations at two different NaCl concentrations. Time series of twist in A) 1.0 and B) 4.4 M NaCl initiated from an ideal B- and C-DNA structure. These simulations were performed with parmbsc1/Mamatkulov-Schwierz force fields.

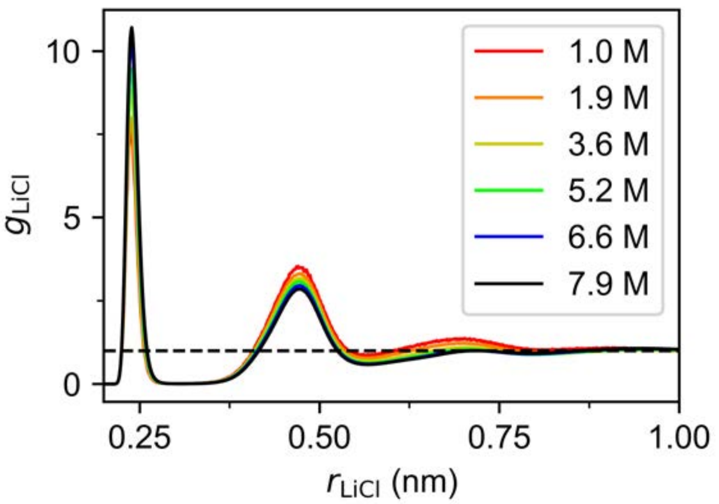

Supplementary Figure S23. Radial distribution functions g_{LiCl} along the distance r_{LiCl} between Li^+ and Cl^- for different concentrations of LiCl in a water box without DNA. The black dashed line indicates unity, which is asymptotically reached at large distances. Simulations used the ion parameters from Mamatkulov-Schwierz in combination with the TIP3P water model. Note that the concentrations in the legend are reported in molarity and correspond to molalities of 1.0, 2.0, 4.0, 6.0, 8.0 and 10.0 m, respectively.

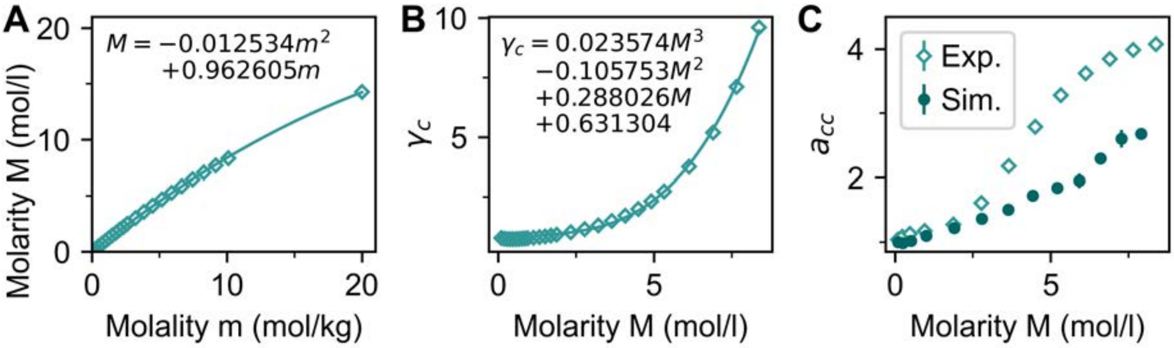

Supplementary Figure S24. Activity coefficients and activity derivatives as function of LiCl concentration in water. A) Molarity plotted against molality for LiCl in water. Data are taken from Ref. (8). The line is a parabolic fit to the data. The equation for the fit is shown as an inset and used for the conversion from molality to molarity. B) Activity coefficient γ_c plotted against concentration (molarity). Points are a selection of values reported in (7). The solid line is a cubic fit to the data with coefficients shown in the inset. C) Activity derivative a_{cc} plotted against concentration. Shown are the results from experiments using numerical differentiation of the cubic fit to the data in panel B (open diamonds) and from MD simulations employing Kirkwood-Buff theory (filled circles). For the simulations, data points correspond to the mean ± SEM from three independent simulation runs.

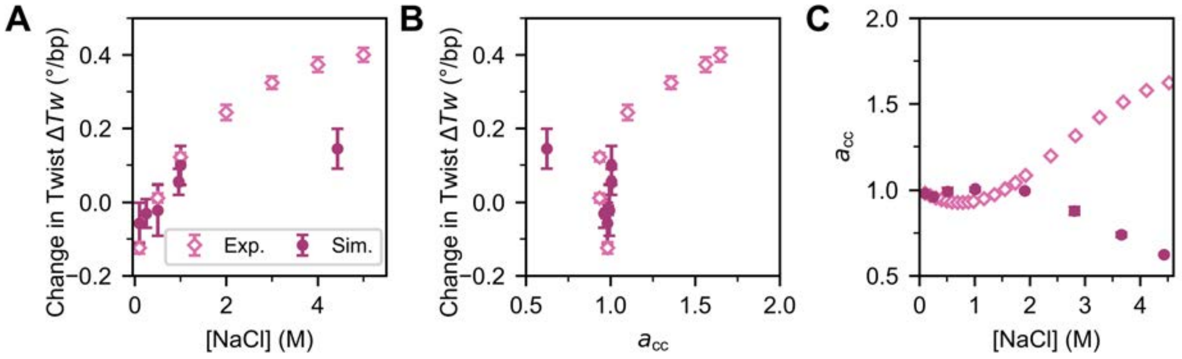

Supplementary Figure S25. Change in twist as function of NaCl concentration and activity derivative. A) Change in twist (ΔTw) as a function of NaCl concentration relative to 0.10 M KCl from experiments and MD simulations with parmbsc1 force field combined with Mamatkulov–Schwierz ion force field, and the TIP3P water model. B) ΔTw as a function of the activity derivative a_{cc} obtained from the a_{cc}-concentration relation shown in panel C from experiments and simulations. C) a_{cc} as a function of the NaCl concentration from experiments and current simulations using Kirkwood-Buff theory. Simulation points and error bars for ΔTw are mainly taken from our previous study (13), except for the lower point at 1 M concentration. That point and the data for a_{cc} are from current simulations indicating the mean and SEM from six independent simulations.

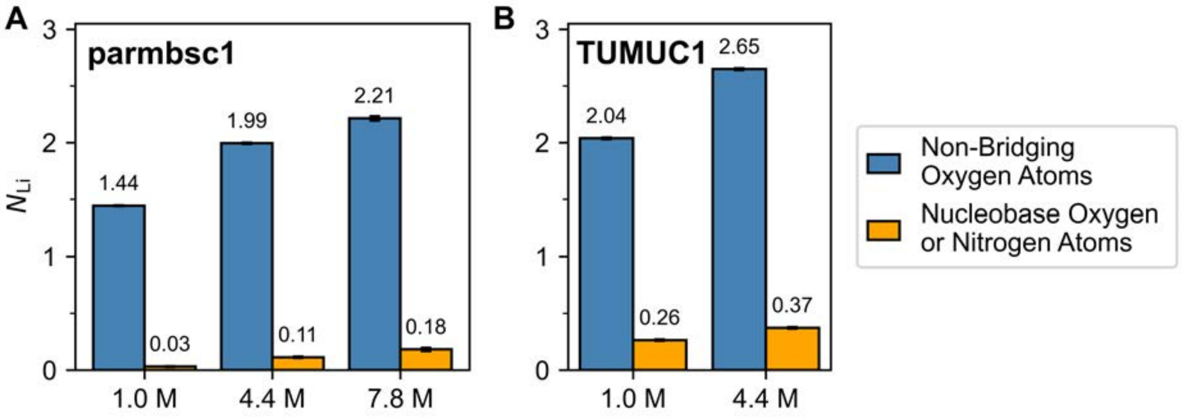

Supplementary Figure S26. Ion adsorption with increasing LiCl concentration for two DNA force fields. Average numbers of Li^+ ions per phosphate group N_{Li} that are within a distance of 0.3 nm to any non-bridging oxygen atom of the phosphate groups or nucleobase oxygen or nitrogen atoms from simulations A) with the parmbsc1 force field or B) with the TUMUC1 force field. Error bars show the standard error from six independent simulations. Note that the reported concentrations are in molarity units and correspond to molalities of 1, 5, and 10 m, respectively.

Supplementary References

1. Gee, M.B., Cox, N.R., Jiao, Y., Bentenitis, N., Weerasinghe, S. and Smith, P.E. (2011) A Kirkwood-Buff Derived Force Field for Aqueous Alkali Halides. *Journal of Chemical Theory and Computation*, **7**, 1369-1380.
2. Mamatkulov, S. and Schwierz, N. (2018) Force fields for monovalent and divalent metal cations in TIP3P water based on thermodynamic and kinetic properties. *The Journal of chemical physics*, **148**, 074504.
3. Kirkwood, J.G. and Buff, F.P. (1951) The Statistical Mechanical Theory of Solutions. I. *The Journal of chemical physics*, **19**, 774-777.
4. Weerasinghe, S. and Smith, P.E. (2003) A Kirkwood–Buff derived force field for sodium chloride in water. *The Journal of chemical physics*, **119**, 11342-11349.
5. Lyubartsev, A.P. and Marčelja, S. (2002) Evaluation of effective ion-ion potentials in aqueous electrolytes. *Phys. Rev. E*, **65**, 041202.
6. Jorgensen, W.L., Chandrasekhar, J., Madura, J.D., Impey, R.W. and Klein, M.L. (1983) Comparison of simple potential functions for simulating liquid water. *The Journal of chemical physics*, **79**, 926-935.
7. Hamer, W.J. and Wu, Y.C. (1972) Osmotic Coefficients and Mean Activity Coefficients of Uni-univalent Electrolytes in Water at 25°C. *Journal of Physical and Chemical Reference Data*, **1**, 1047-1100.
8. Haynes, W.M. (2014) *CRC Handbook of Chemistry and Physics*. CRC Press.
9. Izadi, S., Anandakrishnan, R. and Onufriev, A.V. (2014) Building Water Models: A Different Approach. *The Journal of Physical Chemistry Letters*, **5**, 3863-3871.
10. Kadaoluwa Pathirannahalage, S.P., Meftahi, N., Elbourne, A., Weiss, A.C.G., McConville, C.F., Padua, A., Winkler, D.A., Costa Gomes, M., Greaves, T.L., Le, T.C. *et al.* (2021) Systematic Comparison of the Structural and Dynamic Properties of Commonly Used Water Models for Molecular Dynamics Simulations. *Journal of Chemical Information and Modeling*, **61**, 4521-4536.
11. Horn, H.W., Swope, W.C., Pitera, J.W., Madura, J.D., Dick, T.J., Hura, G.L. and Head-Gordon, T. (2004) Development of an improved four-site water model for biomolecular simulations: TIP4P-Ew. *The Journal of chemical physics*, **120**, 9665-9678.
12. Joung, I.S. and Cheatham, T.E., 3rd. (2008) Determination of alkali and halide monovalent ion parameters for use in explicitly solvated biomolecular simulations. *The journal of physical chemistry*, **112**, 9020-9041.
13. Cruz-León, S., Vanderlinden, W., Müller, P., Forster, T., Staudt, G., Lin, Y.-Y., Lipfert, J. and Schwierz, N. (2022) Twisting DNA by salt. *Nucleic Acids Research*, **50**, 5726-5738.
14. Lu, X.-J. and Olson, W.K. (2008) 3DNA: a versatile, integrated software system for the analysis, rebuilding and visualization of three-dimensional nucleic-acid structures. *Nature Protocols*, **3**, 1213-1227.
15. Lipfert, J., Klijnhout, S. and Dekker, N.H. (2010) Torsional sensing of small-molecule binding using magnetic tweezers. *Nucleic Acids Res*, **38**, 7122-7132.
16. Bunpheng, A., Sakulaue, P., Hirunpinyopas, W., Nueangnoraj, K., Luanwuthi, S. and Iamprasertkun, P. (2023) Revisiting the properties of lithium chloride as “water-in-salt” electrolyte for pouch cell electrochemical capacitors. *Journal of Electroanalytical Chemistry*, **944**, 117645.
17. Golub, G.H. and Van Loan, C.F. (2013) *Matrix computations*. JHU press.
18. Shiratori, T., Goto, S., Sakaguchi, T., Kasai, T., Otsuka, Y., Higashi, K., Makino, K., Takahashi, H. and Komatsu, K. (2021) Singular value decomposition analysis of the secondary structure features contributing to the circular dichroism spectra of model proteins. *Biochemistry and Biophysics Reports*, **28**, 101153.
19. Ivanov, V.I., Minchenkova, L.E., Schyolkina, A.K. and Poletayev, A.I. (1973) Different conformations of double-stranded nucleic acid in solution as revealed by circular dichroism. *Biopolymers*, **12**, 89-110.
